# Botulism in Italy, 1986 to 2015

**DOI:** 10.2807/1560-7917.ES.2017.22.24.30550

**Published:** 2017-06-15

**Authors:** Fabrizio Anniballi, Bruna Auricchio, Alfonsina Fiore, Davide Lonati, Carlo Alessandro Locatelli, Florigio Lista, Silvia Fillo, Giuseppina Mandarino, Dario De Medici

**Affiliations:** 1 National Reference Centre for Botulism, Department of Veterinary Public Health and Food Safety, Istituto Superiore di Sanità (ISS), Rome, Italy; 2 These authors contributed equally to this work; 3 Poison Control Centre and National Toxicology Information Centre, IRCCS Maugeri Foundation Hospital, Pavia, Italy; 4 Histology and Molecular Biology Unit, Section Two, Army Medical and Veterinary Research Centre, Rome, Italy; 5 PENTA - The Joint Laboratory on Models and Methodology to Predict and Manage Large Scale Threats to Public Health, International Affairs Unit, Istituto Superiore di Sanità (ISS), Rome, Italy

**Keywords:** Botulism, Epidemiology, BoNT-producing *Clostridium*, Surveillance, Infectious diseases

## Abstract

Botulism is a rare but severe neuroparalytic disease caused by botulinum toxins. Because of its high potential impact on public health, botulism is a closely monitored communicable disease in Europe. In Italy, which has one of the highest incidence rates in Europe (0.03 cases per 100,000 population), botulism is monitored through a case-based passive surveillance system: the front-line physician who diagnoses a suspected case must notify the Local Health Units immediately, and the Ministry of Health's office within 12 hours. From 1986 to 2015, 466 confirmed cases of botulism were recorded in Italy (of 1,257 suspected cases). Of these, 421 were food-borne (the most frequently seen form of botulism due to the consumption of improperly home-canned foods), 36 were infant botulism, which accounts for ca 50% of all these types of cases registered in Europe, six were wound-related and three were due to adult intestinal colonisation. This scenario suggests that stronger efforts should be made towards raising public awareness of the risk of food-borne botulism, especially with respect to home-preserved foods, as well as improving the training of front-line medical personnel, to ensure that a quick and accurate diagnosis of botulism can be made.

## Introduction

Botulinum neurotoxins (BoNTs) are the most potent poisons known [1,2]. Due to their formidable potency BoNTs can be used as biological weapons. Because of the potentially high public health impact, the occurrence of botulism cases and outbreaks is closely monitored. All seven antigenic variants of BoNTs identified to date (A to G) act on presynaptic neurons, blocking the release of the neurotransmitter acetylcholine in the neuromuscular junctions. Botulism onset may include a prodromal phase, characterised by gastrointestinal discomfort and anticholinergic symptoms such as xerostomia (dry mouth), while the typical final syndrome consists of a symmetrical cranial nerve palsy, followed by symmetrical descending flaccid paralysis of both voluntary and autonomic muscles [1-4]. The diagnosis of botulism is based on clinical examination and confirmed by laboratory testing [1,2]. The treatment of the disease includes administration of botulinum antitoxin serum and, when required, support of respiratory function [1,2]. Different formulations containing varying quantities and combinations of specific types of antitoxins are used worldwide. Serum available in Italy is distributed by the Ministry of Health (MoH) and consists of 250 ml of trivalent equine antitoxin, protecting against BoNTs of type A, B, and E. The recommended dose is two vials; each vial contains 187,500 IU against-BoNT/A, 125,000 IU against-BoNT/B and 12,500 IU against-BoNT/E [2].

BoNTs are produced *Clostridium botulinum* and also by rare strains of *Clostridium baratii* and *Clostridium butyricum* [2-4].

To date, six forms of botulism are recognised and classified according to the modality of exposure to the toxin [2,5]: (i) Food-borne botulism occurs after the ingestion of preformed BoNT in food [6]; both (ii) infant botulism and (iii) adult intestinal colonisation (also collectively referred to as intestinal toxaemia botulism) are caused by the ability of spores to germinate in the colon, producing BoNTs in situ; (iv) wound botulism is the consequence of in vivo toxinogenesis of *C. botulinum* spores contaminating an injury [1]; (v) iatrogenic botulism is a complication of the treatment with BoNTs for therapeutic or cosmetic use [1] and (vi) inhalation botulism, results from accidental or deliberate release of aerosolised toxins [1,5]. Iatrogenic and inhalation botulism are the two non-naturally occurring forms [5].

Although botulism is a rare disease worldwide, the Italian incidence rate is one of the highest in Europe, ranging between 0.02 and 0.04 cases per 100,000 population from 2006 to 2010 [7,8]. All of the naturally occurring forms of botulism are represented, and although food-borne botulism is the prevalent form, the number of infant botulism cases reported in Italy represents ca 50% of all European cases [8,9].

Here we provide detailed information on epidemiological features of clinical cases and outbreaks of botulism in Italy from 1986 to 2015.

## Material and methods

### Surveillance system

In Italy, the MoH included botulism as a notifiable disease in 1975, and it became a Class I disease, i.e. one requiring immediate reporting, in 1990 [10]. The current reporting system requires the front-line physician to notify Local Health Units immediately of all suspected cases, and then proceed with notifying the Regions, the MoH and the Istituto Superiore di Sanità (ISS), National Reference Centre for Botulism (NRCB) and National Centre for Epidemiology, Surveillance and Health Promotion within 12 hours of their initial formulation of clinical suspicion [11]. The NRCB and other accredited local laboratories receive clinical samples for laboratory testing the results of which are sent to the physician in charge of the case and the MoH. The MoH, in collaboration with the NRCB, collects all data and transmits them to the European Centre for Disease Prevention and Control (ECDC). Pavia Poison Control Centre (PPCC) provides expert clinical advice for acute clinical management, including indications for antitoxin administration, and performs a targeted clinical follow-up for possible late complications or adverse effects (e.g. serum sickness).

### Case definitions

From 1975 to 1991, no standard case definition of botulism was used in Italy, while from 1991 to 1996 the MoH adopted the case definitions published by the United States Centers for Disease Control and Prevention (CDC) [12]. According to these, only laboratory-confirmed cases were classified and reported. On 1 July 1996, the MoH amended the definition publishing the Circular Number 9 laying down the ‘Misure di prevenzione e controllo delle intossicazioni da botulino’ (measures for prevention and control of botulism) [13]. In 2002, the European Commission adopted Decision 2002/253/EC aimed at standardising case definitions of communicable diseases among Member States [14]. Unlike the previous definitions used in Italy, the first European case definitions made no distinction among the different forms of botulism, although confirmed cases (defined as clinically compatible cases confirmed in the laboratory) were marked as different from probable cases (defined as clinically compatible cases with an epidemiological link) [14]. This latter decision was amended in 2003, 2008 and 2009. The current definitions categorise botulism into two clinical forms: (i) food-borne and wound botulism and (ii) infant botulism; cases are classified as probable or confirmed [15].

For the purposes of this work, the occurrence of a sporadic case or an outbreak of food-borne botulism is defined as an incident.

### Data collection and analysis

Demographic, clinical and epidemiological data were obtained from the patient by the attending physician using a specific notification form, which is sent to both the NRCB and the MoH. Additional epidemiological investigations may be performed by the Department of Hygiene and Public Health and Local Health Units and additional information collected by the NRCB during phone interviews with patients and relatives and/or by the PPCC during patient clinical follow up. All these data, together with the microbiological and laboratory analyses results, are collected and stored in a specific database by the NRCB, which transmits all data to the MoH annually.

Statistical analysis was performed using Microsoft Excel 2010 (Microsoft Corp., US) and Prism version 6.03 (GraphPad Software, Inc., La Jolla, CA, US) by univariate analysis with chi-squared and t tests, as appropriate. A result of p < 0.5 was considered to indicate statistical significance.

### Laboratory investigations

Clinical specimens for laboratory confirmation are taken by the attending clinician as soon as a diagnosis of botulism is suspected, and are sent for testing as soon as possible. Arrangements for testing leftover food are also made as a matter of urgency. If necessary, other foods are collected and sent for testing during any further epidemiological investigations by Local Health Units (Departments of Hygiene and Public Health). The NRCB carries out at least 90% of laboratory diagnosis performed in Italy, using analytical methods accredited according to ISO 17025 [16]. Detection of BoNTs was carried out by mouse bioassay, while detection of BoNT-producing *Clostridium* was carried out through multiplex real-time PCR developed and validated by the NRCB [17,18].

## Results

From 1986 to 2015 a total of 1,257 suspected cases of botulism were notified to the NRCB. Of these, 466 cases were laboratory-confirmed (Table 1).

**Table 1:** Number and percentage of suspected and laboratory-confirmed cases by type of botulism, Italy, 1986–2015

Type of botulism	Suspected cases			Laboratory-confirmed cases		
	Number	%	95% CI	Number	%	95% CI
Food-borne	1,173	93.3	91.9 to 94.7	421	90.4	87.7 to 93.0
Infant	70	5.6	4.3 to 6.8	36	7.7	5.3 to 10.1
Wound	9	0.7	0.2 to 1.2	6	1.3	0.3 to 2.3
Adult intestinal colonisation	5	0.4	0.0 to 0.7	3	0.6	−0.1 to 1.4
**Total**	**1,257**	**100**	**NA**	**466**	**100**	**NA**

CI: confidence interval; NA: not applicable.

Male patients represented 51.7% of the confirmed cases (241/466) while females represented 45.7% (213/466) of cases. For the 12 remaining patients, sex was not reported.

**Figure 1:**
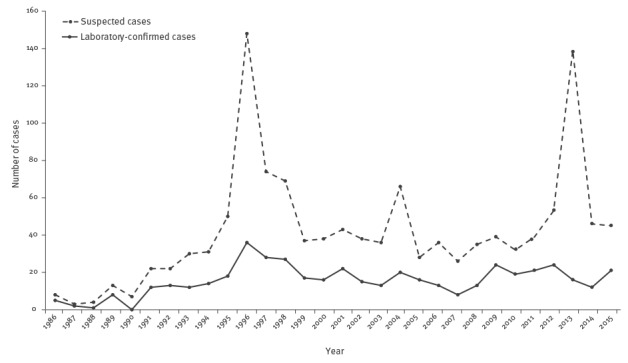
Suspected (n=1,254) and laboratory-confirmed cases (n=466) of botulism, Italy, 1986–2015

**Figure 2:**
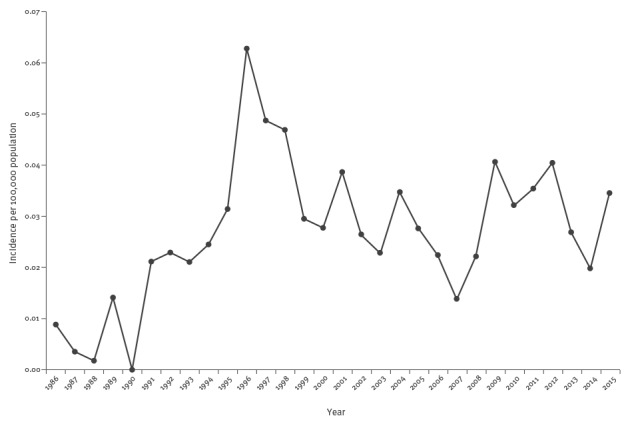
Annual incidence of botulism per 100,000 population, Italy, 1986–2015 (n=1,254)

Overall, the number of both notified and confirmed cases increased from 1986 to 1994, with an average of 40 notifications and 15 confirmations per year. The largest peak in notification of suspected cases was observed in 1996 as a consequence of four outbreaks due to commercial foods (mascarpone cheese and olives), and in 2013 as a consequence of a suspected outbreak due to commercial pesto sauce (Figure 1). The average annual incidence during the entire surveillance period was 0.03 per 100,000 population (range: 0.00–0.06), see Figure 2.

Ninety-six percent (316/330) of the laboratory-confirmed incidents were due to the neurotoxin produced by proteolytic strains of *C. botulinum*. Type B toxin was implicated in 79.1% (261/330) of all confirmed incidents, followed by type A (9.7%, 32/330), and type F, Ab and Bf toxins, accounted for 0.3% (1/330), 1.5% (5/330) and 0.6% (2/330) of the total incidents, respectively.

No clear seasonal pattern was observed for food-borne botulism, although during the holiday periods (Christmas, Easter and August holidays) the number of suspected cases usually increased. Conversely, a seasonal pattern was detected for infant botulism: 11 of 36 confirmed cases occurred in April, an observation which has yet to be explained.

### Food-borne botulism

From 1986 to 2015, 285 laboratory-confirmed incidents involving a total of 421 persons were recorded. The mean number of cases per incident was 1.5 (range: 1–16 cases per incident).

Most confirmed incidents, involving 241 persons in total, originated in rural areas of central and southern regions of Italy (Table 2), and in particular in Campania (50/285, 17.5%), Puglia (40/285, 14.0%), Lazio (31/285, 10.9%), Sicilia (22/285, 7.7%), and Calabria (21/285, 7.4%).

**Table 2:** Number of laboratory confirmed cases by regions and type of botulism, Italy, 1986–2015 (n=466)

Region	Food-borne botulism	Infant botulism	Wound botulism	Adult intestinal colonisation botulism
Piemonte	20	0	1	1
Valle d’Aosta	0	0	0	0
Lombardia	21	5	0	0
Trentino Alto Adige	4	0	0	0
Veneto	19	4	0	1
Friuli Venezia Giulia	15	2	0	0
Liguria	14	0	0	0
Emilia Romagna	28	2	0	1
Toscana	4	0	0	0
Marche	6	0	0	0
Umbria	9	1	0	0
Lazio	43	11	1	0
Sardegna	10	0	0	0
Abruzzo	11	0	1	0
Molise	4	0	0	0
Campania	71	6	0	0
Basilicata	15	0	0	0
Puglia	58	4	1	0
Calabria	39	1	0	0
Sicilia	30	0	2	0
**Total**	**421**	**36**	**6**	**3**

In these areas, many people still maintain the tradition of preparing home-canned foods, due to the low cost and wide availability of raw food materials. In the 10 years to 2015, an increasing number of cases were reported in Emilia Romagna, Lombardia and Piemonte (northern Italy). More than 90% of these cases involved university students (mostly male) of southern Italian origin, who had consumed homemade canned food prepared by their mothers.

The median age of cases was 43 years (range: 0–93 years) during the entire surveillance period: 49.2% of patients (207/421) were in the 25–65 years age group, 51.3% (216/421) were male.

#### Hospitalisation and clinical symptoms

Although all patients were admitted to hospital, length of hospitalisation was unknown because the reporting system did not record this information or the medical follow up. As reported in Table 3, dysphagia was the most common clinical symptom (304/421, 70.6% of cases).

**Table 3:** Clinical signs and symptoms reported by patients with food-borne botulism, Italy, 1986–2015 (n=421)

Clinical sign/symptom	Number of cases	% of cases
Headache	28	6.6
Double vision	298	70.6
Drooping upper eyelid	43	10.2
Dilation of the pupil	88	20.9
Difficulty in swallowing	304	72.0
Dry mouth	278	65.9
Facial palsy	28	6.6
Respiratory failure	75	17.8
Constipation	209	49.5
Nausea	145	34.4
Vomiting	157	37.2
Abdominal pain	6	1.4
Diarrhoea	40	9.5
Urinary retention	20	4.7
Coma	9	2.1
Death	17	4.0

Usually, patients presented mild symptomatology, with a clinical picture including diplopia (double vision), dysphagia (difficulty in swallowing), and dry mouth in ca 50% of the cases. Respiratory failure was reported in 17.8% (75/421) of patients. As for gastrointestinal symptoms, vomiting was reported by 37.3% (157/421), nausea by 34.4% (145/421), and diarrhoea by 9.5% (40/421) of the patients. A total of 16 deaths were recorded, giving a case-fatality rate of 3.8% (16/421), with four of the deaths occurring in elderly patients aged over 80 years who lived alone. The highest numbers of deaths were seen in 1996 and 2005, with three and four deaths respectively. Type B (11 cases), A (4 cases), and Ab (1 case) neurotoxins were detected in clinical specimens from the cases who died.

#### Botulinum antitoxin administration

Data on botulinum antitoxin administration were available from 1995 onwards, for 300 patients: Of these, 162 (54.0%) received the antitoxin. Although the manufacturer-recommended dose is two vials, 71/162 patients (43.8% of all those who received the antitoxin) were treated using different dosages. In particular, 46 patients received half the dosage recommended by the producer. No cases of adverse reactions to the antitoxins have been reported through the official botulism surveillance system; however, the Pavia Poison Control Centre reported that of 59 patients treated from 2007 to 2015, six showed adverse reactions.

#### Laboratory investigations

Serum was tested for 65.3% of patients (275/421) of confirmed food-borne cases and resulted positive only for 20.4% of them (56/275). The remaining cases were confirmed by direct detection of toxins in faecal samples (52 patients) or foods (159 patients). A further 154 food-borne cases presenting with the characteristic clinical picture of botulism were laboratory-confirmed, with BoNT-producing *Clostridium* isolated from faecal samples.

#### Food items involved

Food was identified as the transmission vehicle either by laboratory testing or following epidemiological investigations in 41.4% (118/285) and 30.7% of all confirmed incidents (86/285), respectively. A total of 80.5% (95/118 incidents, involving 143 persons) of food items linked to confirmed incidents consisted of homemade canned food, while the remaining 19.5% (23/118) was commercial food. Only one outbreak was connected to restaurant-canned green olives. Vegetables canned in oil and in brine/water were associated with 43.2% (51/118) and 28.8% (34/118), respectively, of laboratory-confirmed incidents. Other types of food related to laboratory-confirmed incidents were home-bottled tuna (9/118, 7.6%), ham (7/118, 5.9%), home-bottled meat (7/118, 5.9%), salami/sausages (5/118, 4.2%), cheese (3/118, 2.5%) and tofu and seitan (2/118, 1.7%). Among vegetables, the most frequent products involved in cases or outbreaks were mushrooms in oil (27 incidents involving 40 people), olives (eight incidents, 19 cases) and turnip tops (eight incidents, 17 cases). The most common food not analysed in the laboratory but connected to incidents via epidemiological investigations was vegetables in oil (51/86, 59.3%) and vegetables in water/brine (21/86, 24.4%). Of these, mushrooms were linked to 19 incidents (21 patients), leafy vegetables to eight incidents (eight patients) and peppers to six incidents (six patients). In Italy, meat products are rarely linked to botulism. Of 18 incidents connected to these products, 16 were due to consumption of home-prepared foods. From 1986 to 2000 these home-prepared products were most often improperly preserved ham and sausages, while from 2001 to 2015 to the most representative infection vehicle was home-bottled meat brought in to Italy by Eastern European workers. Interestingly, the latter exclusively involved males and often occurred after visits home to native countries for Christmas when it is common to return with traditional home-bottled foods. A combination of improper preparation and storage of jars were at the basis of these incidents. Regarding fish products, home-canned tuna was the most common food linked to confirmed incidents. Cheese or dairy products were seldom associated with confirmed incidents, even though the most well-known botulism incident ever to occur in Italy was related to mascarpone cheese [19].

### Intestinal toxaemia botulism

From 1986 to 2015 only three cases of adult intestinal colonisation botulism were reported in Italy, in two males (a 9-year-old boy and a man in his mid-50s) and one female (19 years old). *Clostridium butyricum* capable of producing type E toxin was recovered in the faecal samples of the 9-year-old male and the 19-year-old female, while *C. botulinum* type A was recovered from the faecal samples of the other patient. The patients whose botulism was due to *C. butyricum* type E had serious gastrointestinal symptoms with acute pain, and both underwent surgery for suspected appendicitis. The neurological symptomatology was initially mild, but worsened rapidly after the surgery and both patients required mechanical ventilation. During surgery, both patients were found to have a Meckel’s diverticulum. Following the surgery the male patient was treated with rifampicin while the female patient with ceftazimide. The third patient, a man in his mid-50s, was admitted to hospital with diplopia, dysphagia, nausea and vomiting with no fever. Thirty days before the hospitalisation he had undergone heart surgery and received postsurgical antibiotic therapy consisting of ceftriazone for 2 days. Approximately 1 month after heart surgery the neurological symptoms persisted and *C. botulinum* type A was recovered by stool cultures. As potential suspected food items could not be identified as being consumed by the patient and yet *C. botulinum* persisted in the intestinal tract for at least 6 weeks, adult intestinal colonisation botulism was diagnosed.

### Infant botulism

From 1986 to 2015, a total of 36 cases of infant botulism (17 boys and 19 girls) were laboratory-confirmed. In all 36 cases, the patients were hospitalised: 20 infants received parenteral feeding and 13 required mechanical ventilation because respiratory failure had occurred. The length of the hospital stay was known only for 27 infants, reporting an average of 33.6 days (median = 28.0 days). One boy stayed in hospital for over 150 days. The average age at hospital admission was 16.2 weeks (range: 4–33 weeks). Before symptom onset, all infants had been in good health: 26 infants had been breast-fed, two had been formula-fed, and eight had been both breast- and formula-fed; 12 of them had started weaning. Honey consumption and herbal infusion was reported for 20 and nine infants, respectively. Nineteen patients received broad-spectrum antibiotics because severe infection was suspected before an infant botulism diagnosis was made. Nine infants were treated using equine botulinum antitoxin to avoid worsening of the symptoms. The first four patients received an amount of 40 ml/kg, 23 ml/kg, 16 ml/kg and 10 ml/kg per body weight of equine-derived antitoxins (see food-borne botulism section), respectively. The others received a dosage 10 ml/kg per body weight. No adverse effects to equine antitoxin were recorded for any patient.

All cases were laboratory confirmed and neurotoxigenic strains were isolated as *C. botulinum* type B in the faeces of 26 infants, *C. botulinum* type A in 5 and type E *C. butyricum* in three other cases. The remaining two cases were due to type Ab and Bf *C. botulinum*, respectively. Faecal specimens were tested for BoNT in only 23 cases (for the others, only rectal swabs were tested) and gave positive results in 17 infants (13 type B, 3 type A, and 1 type E). Due to the low amount of stool received for testing, a spore count was performed on only 12 samples, obtaining a range from 21 to 1,000,000 spores per gram. The testing for persistence of spores in the intestinal tract of patients was routinely performed by the NRCB, which collected samples every 2 days. In these 36 patients, spores persisted for an average of 18.5 days (range: 7–97 days). For 23 infants, food (honey and herbal infusion) and other environmental samples were collected from their homes and examined for BoNT-producing *Clostridium*. Five honey samples were positive but the strain isolated was of a different toxin type to that isolated from the infants.

### Wound botulism

In Italy, the first case of wound botulism was diagnosed based on clinical observation in 1976. From 1986 to 2015, six cases were reported. Except for one case involving a drug user, all remaining cases were due to traumatic injuries (accidental falls or other accidents at work). All patients were adults (mean age 43.7 years old, range: 24–61 years), five were male and one was female. Neurological symptoms occurred a mean 10.3 days after the injury (range: 7–17 days), including ptosis (drooping of the upper eyelid), mydriasis (pupil dilation), diplopia and dysphagia in all seven patients, constipation and respiratory failure in three cases, and fever in two cases. Antibiotic therapy was administered to six patients: ampicillin combined with cephalosporin, netilmicin, amoxicillin and clavulanate, ciprofloxacin, metronidazole, ertapenem, and ceftriaxone were the drugs most frequently used (alone or in combination). Antitoxin therapy was administered to four patients, with adverse reactions noted in only one of them. Finally, hyperbaric oxygen treatment of the injury was used for one patient in 1991.

All cases were laboratory-confirmed by means of BoNTs detection in serum (5 patients) and through the isolation of BoNT-producing strains from wound exudate. Five of the six cases were due to type B botulism; in the remaining case, the type of toxin was not determined.

## Discussion

Botulism remains a public health concern because of its severity and epidemic potential, as well as its possible use as a biological weapon. In Italy, food-borne botulism due to traditional home-canned food still represents a public health challenge mainly in the southern regions, where improper canning procedures are the primary reason for the occurrence of cases and outbreaks. On the contrary, cases due to refrigerated processed food with extended durability are concentrated in northern regions, where home-canning of foods is less common [10]. Since improper storage conditions seem to be the most frequent cause, permitting BoNT-producing *Clostridium* growth and toxinogenesis, it is safe to assume that continuous education and information to consumers on the best hygienic practices and on the correct home-canning procedures are the most effective preventive measures.

Adult intestinal colonisation botulism is very rare, both in Italy and worldwide. As reported by Fenicia and colleagues [20], abnormality of the gastrointestinal tract following inflammatory intestinal diseases or surgery, and alterations produced by broad-spectrum antibiotics in the endogenous microbiota, which act as the natural barrier to intestinal colonisation, are the only predisposing factor recognised to date. However, the patients from whom *C. butyricum* type E was recovered had Meckel’s diverticulum, which may be considered as a possible predisposing factor for this form of botulism.

With respect to infant botulism, it is important to note that the relatively high number of cases reported in Italy are concentrated in a few paediatric hospitals in a few regions (Table 2), thanks to physicians who have acquired high awareness of this form of botulism and are able to promptly formulate clinical suspicion. Greater efforts have to be made to improve awareness among physicians operating in small-town hospitals. In fact, in many cases the diagnosis of infant botulism was formulated only once the patients were transferred from small, local hospitals to the paediatric hospitals mentioned above. Often the diagnosis of infant botulism is closely related to honey consumption, although other sources of contamination have also been identified [9].

Considerable efforts are needed to improve diagnostic skills in order to identify wound botulism in drug users. Indeed, diagnosis of this rare form of botulism is made more difficult by some drug effects, which can mask neurological symptoms at their onset. The incidence of only a single confirmed case is representative of the difficulties encountered by the physicians in the diagnosis of this form of botulism.

As revealed by the high number of laboratory-confirmed infant botulism cases in Italy, and by the low number of food-borne cases constituting outbreaks (mean cases per outbreaks = 1.5; range: 1–16 cases), the Italian botulism surveillance system demonstrates the ability to recognise and diagnose botulism and implement appropriate control procedures. A synergistic combination of epidemiological investigations and the ability of designated laboratories to detect the causative organism and transmission vehicles provides an effective way to tackle botulism emergencies. However, greater efforts must be put into reaching out to the public in order to increase awareness of food-borne botulism risks and promote correct home-preservation and canning practices. At the same time there is a need for increased awareness among front line medical professionals about the different forms of botulism so that clinical suspicion is considered early, which is essential for prompt diagnosis and treatment of patients.
